# Evidence-based care pathways for people with dementia and neurodegenerative disorders in Europe: a systematic review of models, integration of digital technologies, and quality indicators

**DOI:** 10.3389/fmed.2026.1774318

**Published:** 2026-06-29

**Authors:** Maria Isabel Cardona, Parthena Nena Gakoudis, Jūratė Macijauskienė, Jochen René Thyrian

**Affiliations:** 1 German Center for Neurodegenerative Diseases (DZNE), Rostock/Greifswald, Germany; 2 Department of Geriatrics, Lithuanian University of Health Sciences (LSMU) Kaunas Hospital, Kaunas, Lithuania; 3 Institute for Community Medicine, University Medicine Greifswald, Greifswald, Germany

**Keywords:** care pathways, dementia, integrated care, neurodegenerative disorders, systematic review

## Abstract

Dementia and other neurodegenerative disorders place growing pressure on European health systems, yet evidence on how care pathways are structured, implemented, and evaluated remains fragmented. This systematic review synthesizes European evidence on dementia and neurodegenerative care pathways and develops an evidence-informed conceptual synthesis capturing core functions, domains of care delivery, and cross-cutting implementation principles. The review followed PRISMA 2020 guidelines and was registered in PROSPERO (CRD420251020962). Five databases (PubMed/MEDLINE, Embase, CINAHL, Web of Science, and the Cochrane Library) and gray literature sources were searched for studies published in the last 10 years (to June 2025) examining structured care pathways. Risk of bias was assessed using design-specific tools (RoB-2, ROBINS-I, MMAT), and findings were synthesized narratively due to substantial heterogeneity. Eighty-one studies from 11 European countries and multinational initiatives were included. Reported pathways varied widely in scope, structure, and implementation. Most focused on early-stage processes (referral, diagnosis, and initial post-diagnostic support), while later-stage elements (long-term management, advance care planning, and palliative care) were less frequently represented in the literature. Across studies, a consistent functional core was recurrently reported, including care coordination, multidisciplinary teamwork, case management, caregiver involvement, and structured follow-up. Surrounding domains, such as workforce training, digital and assistive technologies, and quality measurement, were described with variable levels of formalization. Findings reflect patterns reported in the literature rather than a direct representation of routine care across European health systems. Substantial heterogeneity in study designs, reporting practices, and pathway descriptions limited comparability across contexts. This review identifies common organizational functions underlying dementia care pathways and proposes an evidence-informed conceptual framework to support interpretation, comparison, and future development of more integrated and person-centered care models.

## 1 Introduction

Dementia and other neurodegenerative disorders, such as Alzheimer’s disease, Parkinson’s disease, and frontotemporal dementia, show a rising prevalence in Europe, affecting millions of people and generating substantial social, economic, and health system burden (1, 2). The progressive decline in cognitive, behavioral, and functional abilities associated with these conditions can undermine independence and quality of life, while families and informal caregivers may face increasing care demands and psychological stress over prolonged disease trajectories (1, 2). In parallel, European health systems experience sustained pressure through increased hospitalisations, long-term care needs, workforce demands, and rising costs (3–5). Addressing this complex and evolving burden requires integrated, multidisciplinary, and person-centered models of care capable of supporting medical management, psychosocial needs, and functional independence for both individuals living with neurodegenerative disorders and their caregivers (2). Although conditions such as dementia, Parkinson’s disease, motor neuron diseases, and progressive ataxias differ substantially in their clinical manifestations and disease-specific management needs, they share several organizational challenges, including long-term care coordination, multidisciplinary service provision, caregiver involvement, and continuity of care across disease stages and settings (2, 6, 7). Accordingly, the present review focuses on shared organizational features of care pathways rather than disease-specific clinical management.

Care pathways have been proposed as structured, evidence-based frameworks to guide care from early symptom recognition and diagnosis to treatment, follow-up, and long-term support (8, 9). By defining recommended processes, roles, and standards for diagnosis, treatment, monitoring, and coordination across services, care pathways aim to improve continuity of care, facilitate timely interventions, and strengthen communication within multidisciplinary teams and with patients and families (9). More recently, the integration of digital tools, such as telehealth, electronic care plans, remote monitoring, and decision-support systems, has further expanded the potential of care pathways to deliver personalized, scalable, and resource-efficient care across settings (8, 10). When effectively designed and implemented, care pathways can contribute to improved outcomes, more efficient resource use, and reductions in unwarranted variation and inequities across health systems (10).

Despite the growing number of publications on dementia care pathways, the European literature remains fragmented and heterogeneous in how pathways are conceptualized, described, implemented, and evaluated. Previous reviews and comparative analyses have identified persistent gaps in dementia care provision, including discontinuities across care settings, limited service integration, and substantial variation in care structures and access between European countries (11–13). Within the care pathway literature, studies differ widely in scope, focus, and level of integration, with many prioritizing early-stage processes such as diagnostic assessment and initial post-diagnostic support, while giving comparatively limited attention to long-term management, advance care planning, palliative care, and continuity across the full disease trajectory (12, 14). This emphasis within the literature results in descriptions of pathways that are often front-loaded or modular, rather than fully integrated, longitudinal models of care. Recent scoping reviews further indicate that existing pathways lack a shared structure and standardized, comparable outcome and quality indicator sets across settings (14, 15). While this diversity reflects differences in national health system organization, policy priorities, and service contexts, it also poses challenges for continuity, equity, and consistency of care across Europe (3, 12, 16). This gap highlights the need for a comprehensive synthesis that integrates pathway components, organizational functions, and implementation principles across the full disease trajectory.

Beyond descriptive fragmentation, existing reviews and policy analyses point to limited conceptual synthesis integrating pathway components, organizational functions, and implementation principles across the full disease trajectory. Much of the available evidence focuses on discrete elements without articulating how these components interact over time and across settings, resulting in collections of stage-specific practices rather than coherent organizational structures capable of supporting sustained, person- and caregiver centered care (10, 12, 14). This lack of synthesis constrains cross-country learning and limits the development, evaluation, and scaling of integrated and equitable dementia care pathways at the European level (12, 17).

To address these gaps, this systematic review aims to synthesize and critically examine evidence on structured care pathways for people with dementia and other neurodegenerative disorders across Europe. Specifically, the review seeks to: (1) Describe and synthesize the phases and core components of care pathways described in the European literature;

(2) Identify workforce roles, digital and assistive technologies, and implementation features embedded within these pathways;

(3) Examine how outcomes, measurement tools, and quality indicators are used to evaluate pathway performance; and

(4) Develop an evidence-informed conceptual synthesis capturing core organizing functions, key domains of care delivery, and cross-cutting principles shaping pathway implementation across European contexts.

The review is guided by the PICOS framework (Population, Intervention, Comparator, Outcomes, Study design), widely used in systematic reviews (18), which informs the eligibility criteria and scope of the analysis, including the focus on people with dementia and neurodegenerative disorders, structured care pathways, relevant outcomes related to care quality and implementation, and a broad range of study designs.

## 2 Methods

This systematic review was reported in accordance with the PRISMA 2020 reporting guidelines (18). The protocol was prospectively registered in the International Prospective Register of Systematic Reviews (PROSPERO)^1^ under the registration number CRD420251020962, ensuring transparency in the review process. The review question was defined using the PICOS framework, as described in the Introduction, and informed the inclusion and exclusion criteria applied in this review.

### 2.1 Search strategy

The search strategy was designed and piloted iteratively by two researchers with expertise in evidence synthesis. Searches were conducted across several major databases: PubMed/MEDLINE, Embase, CINAHL, Web of Science and the Cochrane Library. All databases were last searched in June 2025. Search strings combined controlled vocabulary and free-text terms referring to dementia and neurodegenerative disorders, structured or integrated care pathways, interprofessional or intersectoral models of care, digital health solutions embedded within service delivery, and outcomes related to quality, coordination, and implementation. The full electronic search strategies for all databases are provided in Supplementary File S1. In addition to database searches, gray literature was systematically explored. Sources included policy documents, reports, and organizational publications from the European Commission, WHO Regional Office for Europe, Alzheimer Europe, and national health agencies. Additional material was identified through collaboration with JADEHealth partners, including national public health institutions and research organizations contributing country-level reports on dementia care pathways.

### 2.2 Eligibility criteria

Studies were eligible if they: (i) focused on people with dementia or neurodegenerative disorders, their informal caregivers, or professionals involved in their care; (ii) examined structured care pathways coordinating services across different levels or sectors of care; (iii) addressed one or more stages of the care pathway (e.g. diagnosis, post-diagnostic support, management, or palliative care), provided these formed part of a broader integrated model; (iv) reported outcomes related to care quality, service integration, efficiency, patient or caregiver experience, or implementation processes; (v) included quantitative, qualitative, mixed-methods, or evaluation study designs; and (vi) were conducted in European countries or were clearly applicable to European health systems. For the purposes of this review, Europe was operationally defined as countries geographically located within the European region, including both European Union (EU) and non-EU countries. Multinational studies were included when their findings were applicable to European healthcare systems. Studies examining the integration of digital technologies were included where these supported care coordination, monitoring, or delivery within a pathway. In this review, neurodegenerative disorders were defined as progressive neurological conditions characterized by neurodegeneration and long-term care needs. The included literature predominantly focused on dementia, including Alzheimer’s disease and related dementias, but also encompassed Parkinson’s disease, motor neuron diseases (including amyotrophic lateral sclerosis), progressive ataxias, and other neurodegenerative conditions where structured care pathways were described.

Peer-reviewed articles, policy reports, and gray literature documents were eligible. Conference abstracts and unpublished manuscripts were excluded unless sufficient methodological and outcome data were available. Only studies published in English were included.

Studies were excluded if they: (i) did not focus on dementia or neurodegenerative disorders; (ii) described isolated interventions without system-level coordination; (iii) focused exclusively on hospital-based or acute care without continuity across settings; (iv) examined standalone digital tools not embedded within a care pathway; or (v) did not report outcomes relevant to the objectives of this review. The review was limited to studies published within the past 10 years to reflect contemporary models of care and the increasing integration of digital technologies within health systems.

### 2.3 Study selection

**FIGURE 1:**
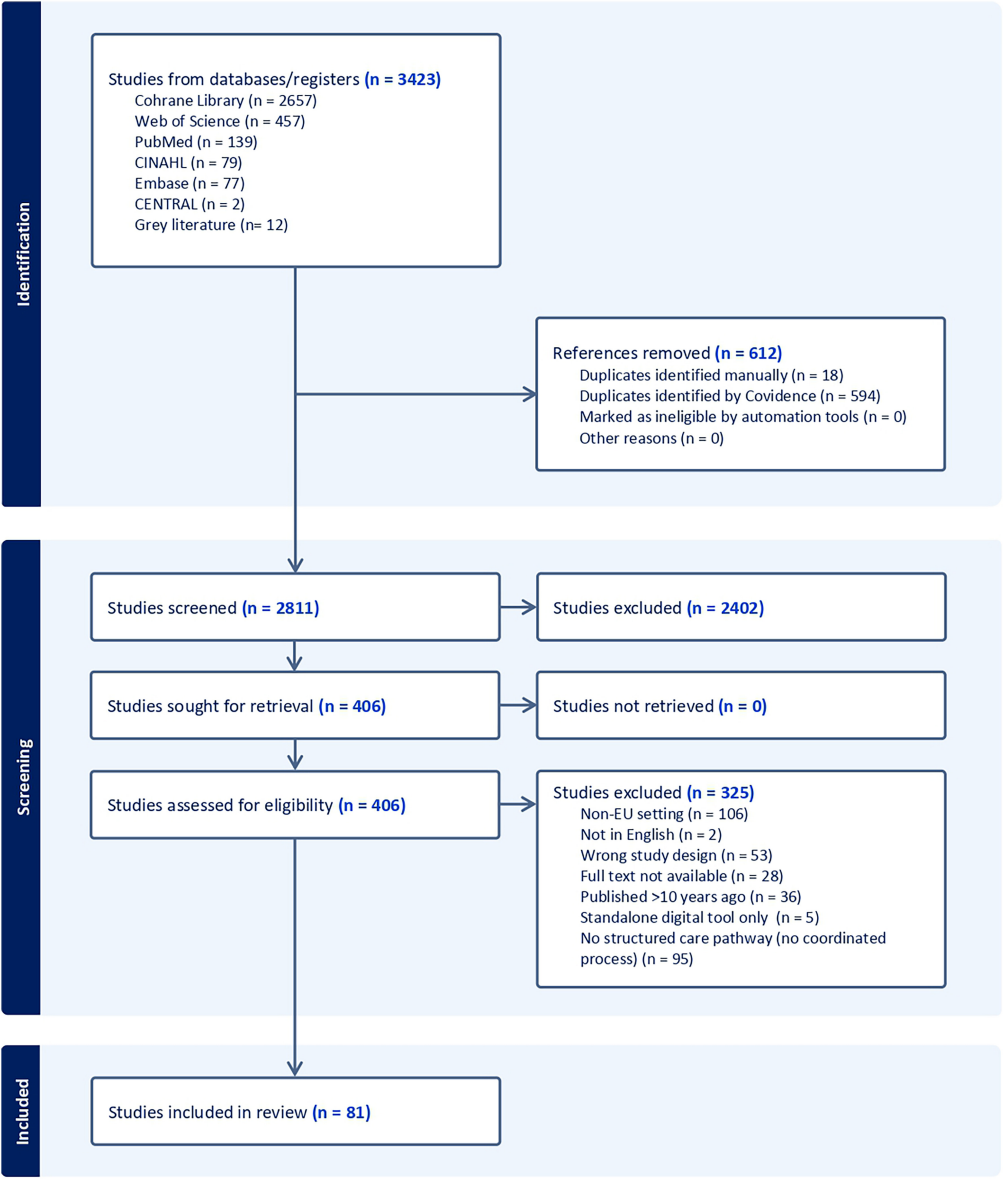
PRISMA flow diagram of study selection for evidence-based care pathways in dementia and neurodegenerative disorders.

All records were screened independently by two reviewers using a two-stage process. In the first stage, titles and abstracts were screened independently by both reviewers. In the second stage, full-text articles were also assessed independently by both reviewers. Disagreements between reviewers were resolved through discussion or, where necessary, by consultation with a third reviewer. The study selection process is summarized in a PRISMA flow diagram (Figure 1).

### 2.4 Data extraction

Data extraction was performed independently by two reviewers using a standardized extraction form developed by the review team based on the review objectives and piloted on a subset of included studies to ensure consistency and completeness. Extracted data included study characteristics, populations and conditions addressed, and the scope of the reported care pathway. Information was also collected on pathway components and phases, workforce roles, training needs, measurement tools, reported outcomes, and quality-related indicators. Where reported, data on the use of digital tools were also extracted. Outcomes were captured at patient, caregiver, and service levels. Any discrepancies between reviewers were resolved through discussion and, where necessary, consultation with a third reviewer. Given the heterogeneity of study designs and reporting, data extraction focused on information relevant to the review objectives. Where multiple outcomes or measures were reported, those most directly aligned with care pathway structure, implementation, and evaluation were prioritized. When data were missing, unclear, or insufficiently reported, no assumptions were made and information was extracted as presented in the original studies.

### 2.5 Quality assessment

The methodological quality and risk of bias of included studies were assessed using design-specific appraisal tools. Randomized controlled trials were evaluated using the Cochrane Risk of Bias 2 (RoB-2) tool (19), which assesses bias across domains including the randomisation process, deviations from intended interventions, missing outcome data, measurement of outcomes, and selection of the reported result. Observational non-randomized studies using ROBINS-I (20), covering domains such as confounding, selection of participants, classification of interventions, deviations from intended interventions, missing data, measurement of outcomes, and selection of reported results. Qualitative, quantitative non-randomized, and mixed-methods studies using the Mixed Methods Appraisal Tool (MMAT) (21), which evaluates methodological quality across study-specific criteria.

All assessments were conducted independently by two reviewers, with disagreements resolved through discussion and consensus. For each study, an overall judgment of methodological quality was derived based on the criteria and guidance provided by each appraisal tool.

Certainty of the synthesized findings was assessed using the GRADE-CERQual approach (22), which evaluates confidence in qualitative and mixed-methods evidence based on four components: methodological limitations, coherence, adequacy of data, and relevance. Confidence in each key finding was graded as high, moderate, low, or very low following established CERQual guidance. The results of the risk of bias and certainty assessments are reported descriptively and summarized in Supplementary File S2, including detailed appraisal tables and CERQual judgements for key synthesized findings.

### 2.6 Data synthesis

**FIGURE 2:**
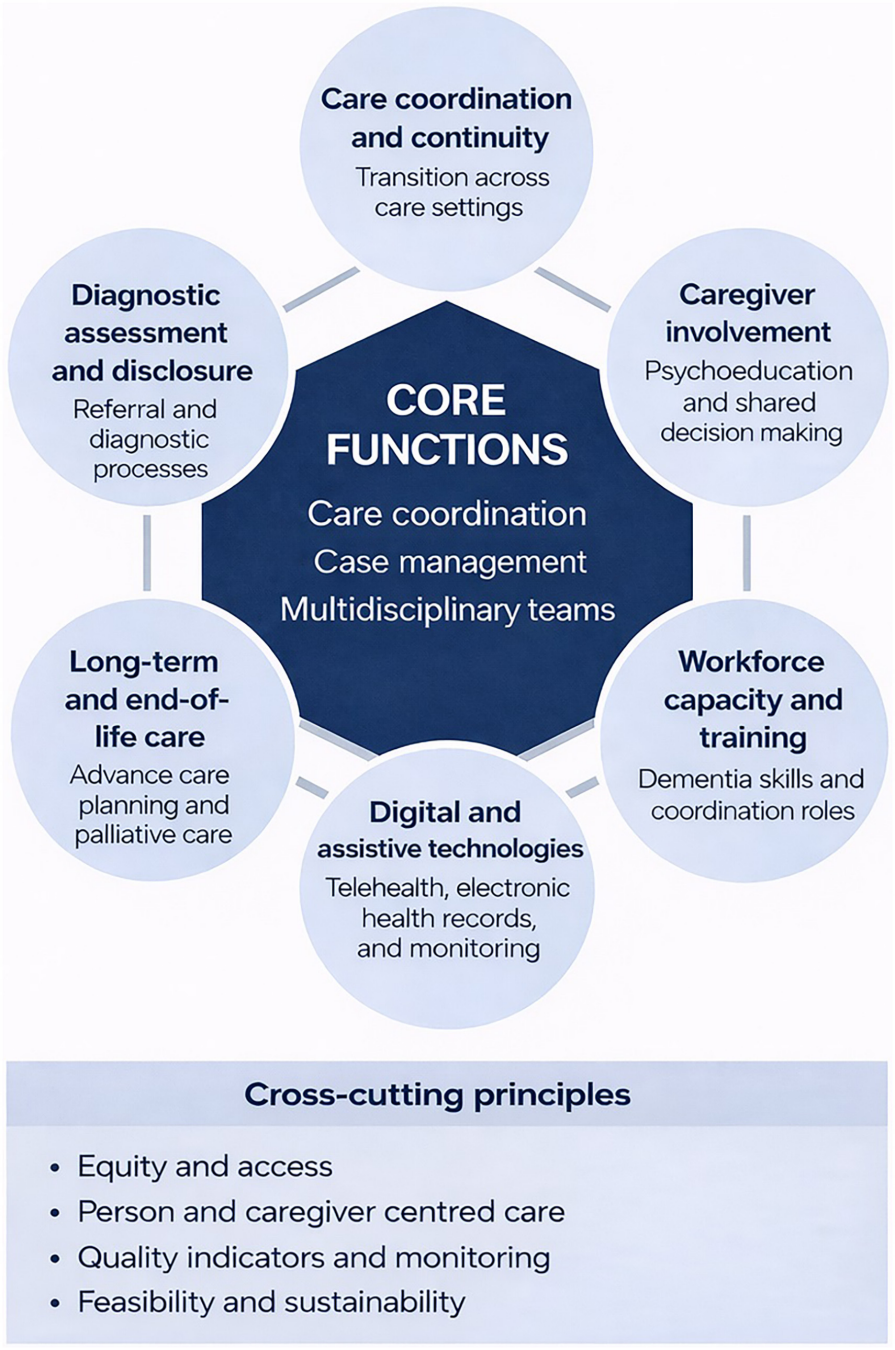
Evidence-informed model of integrated post-diagnostic care pathways for dementia and neurodegenerative disorders.

Given the substantial heterogeneity in study designs, populations, interventions, and outcome measures, quantitative meta-analysis was not appropriate; therefore, a narrative synthesis was conducted. The synthesis followed an iterative, multi-stage approach informed by established guidance for narrative synthesis. First, extracted data were organized and tabulated to enable comparison across studies, including information on pathway structure, components, workforce roles, outcomes, and implementation features. Second, studies were grouped according to key dimensions of interest, including phases of care, types of pathway components, and contextual characteristics related to health system organization. Third, recurring patterns, similarities, and differences across studies were identified through an inductive thematic approach, focusing on care processes, coordination mechanisms, and the role of digital and assistive technologies where reported. Finally, the results of the narrative synthesis informed the development of an evidence-informed conceptual synthesis, derived from the frequency, functional roles, and relationships of components reported across studies. Recurring pathway components were reviewed and compared across studies and grouped according to shared organizational purposes and care functions. Through iterative discussion within the review team, these thematic groupings informed the conceptual synthesis presented in Figure 2, which represents recurring organizational features identified across the included literature rather than a prescriptive or validated care pathway model. This process was conducted collaboratively by the review team to ensure consistency and coherence in the interpretation of findings. Given the variability in reporting across studies, no formal assessment of reporting bias was conducted; however, findings were interpreted with consideration of potential gaps in reporting and uneven evidence availability.

### 2.7 Protocol deviations

Two deviations from the PROSPERO protocol (CRD420251020962) occurred due to limitations in the available evidence. During the data extraction and synthesis stages, the planned HTA-based analysis of digital solutions could not be performed because studies did not report technology-specific outcomes or assessment frameworks. During the synthesis stage, subgroup analyses by country, neurodegenerative condition, and digital integration were not feasible because reporting was highly heterogeneous and disaggregated data were insufficient. These deviations reflect gaps in the evidence rather than changes to the review aims.

## 3 Results

### 3.1 Study selection

The systematic search yielded a total of 3,423 records from electronic databases and gray literature sources. Searches were completed in June 2025, with this cut-off defined a priori to allow completion of screening, data extraction, and synthesis within the project timeline. After removal of duplicates (*n* = 612), 2,811 records remained for title and abstract screening, of which 2402 were excluded. A total of 406 full-text articles were assessed for eligibility, and 81 studies met the inclusion criteria and were included in the review. The study selection process is summarized in the PRISMA flow diagram (Figure 1).

### 3.2 Characteristics of included studies

A total of 81 studies were included in this systematic review. The geographical distribution of the evidence was predominantly European, with the largest proportion of studies conducted in the United Kingdom, followed by multinational studies and Germany. Other countries contributed smaller numbers of studies, including the Netherlands, Italy, France, Sweden, Ireland, and several countries represented by single studies.

With respect to the populations studied, most studies focused on people living with dementia. Parkinson’s disease and motor neuron diseases were less frequently represented, while only a small number of studies addressed other neurodegenerative conditions such as progressive ataxias or unspecified disorders. This distribution indicates that the available evidence base was predominantly dementia-focused. Although the review included a broader range of neurodegenerative disorders, evidence relating to Parkinson’s disease, motor neuron diseases, progressive ataxias, and other conditions was comparatively limited.

In terms of study design, the evidence base was heterogeneous and included randomized controlled trials, qualitative and mixed-methods studies, observational studies, and reports or guideline-based evidence. In addition, a subset of included records consisted of scoping and systematic reviews. These were not treated as primary empirical studies in the synthesis but were used to support the identification, contextualisation, and interpretation of care pathway components across the literature. The distribution of study designs is presented in Table 1.

**TABLE 1:** Characteristics of included studies.

Element	Subcategory/feature	No. of studies (*n* = 81)	Study IDs
Geographic distribution	United Kingdom	26	(47–72)
	Germany	12	(73–84)
	Italy	4	(85–88)
	The Netherlands	6	(89–94)
	Sweden	2	(95, 96)
	France	3	(97–99)
	Norway	1	(100)
	Ireland	2	(101, 102)
	Belgium	1	(103)
	Switzerland	1	(104)
	North Macedonia	1	(105)
	Multinational	22	(6, 12, 14, 36, 88, 106–122)
Population studied	Neurodegenerative disorders (not specified)	4	(65, 70, 94, 98)
	Dementia	61	(12, 14, 15, 36, 47–54, 56, 57, 60, 62–64, 66–69, 71, 72, 74–80, 82–84, 86, 87, 89–97, 100, 102, 104–106, 108, 109, 112–115, 117, 119–122)
	Parkinson’s disease	8	(6, 61, 73, 81, 88, 110, 111, 118)
	Motor neuron diseases (e.g., ALS)	5	(58, 59, 85, 99, 101)
	Progressive ataxias	3	(55, 107, 116)
Study design	Randomized controlled trials (RCTs)	22	(50, 52, 53, 60, 62, 63, 65, 73, 76, 80, 83, 89–92, 96, 97, 100, 103, 105, 110, 117)
	Quantitative/qualitative/mixed /feasibility	27	(48, 51, 56–59, 61, 64, 66, 71, 72, 74, 75, 77–79, 82, 84–86, 88, 94, 102, 104, 114, 116, 122)
	Observational study	5	(68, 95, 98, 101, 119)
	Secondary evidence (scoping/systematic reviews)	14	(6, 12, 14, 15, 69, 106, 108, 111–113, 115, 118, 120, 121)
	Report/guidelines/perspective	13	(36, 47, 49, 54, 55, 67, 70, 81, 87, 93, 99, 107, 109)
Care settings	Primary care	45	(6, 14, 36, 50–53, 56, 64, 67–71, 73–76, 78–80, 82–84, 86, 87, 89, 92, 95, 99–102, 105, 107–110, 113, 116–119, 121, 122)
	Secondary care	45	(6, 14, 36, 47, 49, 50, 55, 58, 60, 63–68, 70, 78, 86–88, 93–95, 97–99, 101, 102, 104, 106, 107, 109, 110, 113, 116, 118–122)
	Tertiary care	20	(6, 36, 59, 62, 65, 73, 87, 88, 93, 94, 99, 101, 102, 106, 107, 109, 116, 118, 119, 121)
	Community-based services	44	(36, 47, 48, 50–58, 67, 69, 71, 73–76, 80, 82–85, 87, 89–92, 96, 105, 108, 110, 113, 114, 116, 118, 121, 122)
	Home-based interventions	30	(6, 52, 53, 56–59, 61, 64, 71, 73–76, 80, 83, 84, 90, 92, 96, 100, 105, 108, 109, 114, 118, 122)
	Long-term care facilities	11	(48, 51, 62, 63, 87, 103, 106, 113, 117, 118, 121)
	Acute hospital	15	(36, 47, 65, 66, 85, 87, 88, 98, 99, 101, 102, 104, 115, 120, 121)

The included studies covered a wide range of care settings. Primary care, secondary care, and community-based services were the most frequently represented, often in combination within integrated care models. Home-based interventions were also common, whereas tertiary care, acute hospital settings, and long-term care facilities were less frequently described.

Overall, the evidence base was concentrated in a limited number of countries and predominantly described pathways spanning primary, secondary, and community care. A detailed overview of study characteristics is provided in Table 1. Given the number and heterogeneity of included studies, full study-level characteristics and extracted data are reported in Supplementary File S3.

### 3.3 Risk of bias and methodological quality

A summary of risk-of-bias and methodological quality judgments across study designs is presented in Table 2. Domain-level and overall assessments for individual studies, together with detailed appraisal notes and CERQual judgments for synthesized findings, are provided in Supplementary File S2.

**TABLE 2:** Studies quality appraisals.

Study design	Tool used	Number of studies	Overall quality/risk of bias
RCTs	RoB-2	22	Low risk of bias: 2/22 (9%); Some concerns: 10/22 (45%); High risk of bias: 10/22 (45%). The most frequent sources of bias were outcome measurement (Domain 4) due to subjective or proxy-reported primary outcomes in non-blinded trials, as well as missing outcome data and attrition (Domains 3 and 5), particularly in cluster-randomized and pragmatic designs.
Observational studies	ROBINS-I	5	Moderate risk of bias: 2/5 (40%); Serious risk of bias: 3/5 (60%). The main sources of bias were confounding and selection bias, particularly in retrospective and cross-sectional designs. Outcome measurement was generally low risk where registry or administrative data were used. These studies were primarily descriptive or associative and provide limited support for causal inference.
Qualitative/quantitative/ mixed-methods	MMAT	25 (of 27)	High methodological quality: 16/25 (64%); Moderate quality: 9/25 (36%); Low quality: 0. Most studies met the majority of MMAT criteria. Qualitative studies consistently demonstrated strong methodological rigor. Mixed-methods and quantitative non-randomized studies were mainly limited by feasibility-oriented designs, partial integration of data, small samples, or lack of comparators rather than major methodological flaws.

Among the 22 randomized controlled trials, assessed using the Cochrane RoB-2 tool, methodological quality was variable. While a small proportion of trials were judged to be at low risk of bias, approximately half showed some concerns and nearly half were rated as high risk of bias. The most frequent limitations related to outcome measurement, particularly the use of subjective or proxy-reported primary outcomes in non-blinded trials, as well as missing outcome data and attrition, especially in cluster-randomized and pragmatic study designs.

The five observational studies assessed using ROBINS-I were rated as having moderate to serious risk of bias. The primary sources of bias were confounding and selection bias, reflecting the non-randomized, retrospective, or cross-sectional nature of these studies. Outcome measurement was generally robust where registry or administrative data were used; however, these studies were mainly descriptive or associative and therefore provide limited support for causal inference.

Of the 27 qualitative, quantitative non-randomized, and mixed-methods studies, 25 were eligible for MMAT assessment; two could not be appraised using MMAT because they did not report sufficient empirical methodological detail or fell outside the scope of the tool. Most of these 25 appraised studies demonstrated high or moderate methodological quality. Qualitative studies consistently showed strong methodological rigor. Mixed-methods and quantitative non-randomized studies were primarily limited by feasibility-oriented designs, partial integration of data, small sample sizes, or lack of comparators, rather than by major methodological weaknesses.

Given the heterogeneity in study designs, populations, and outcomes, no formal investigation of sources of heterogeneity was undertaken; instead, heterogeneity was considered descriptively in the narrative synthesis. Overall, the body of evidence showed substantial heterogeneity in study design and methodological quality. While several studies had limitations relevant to causal inference and effectiveness estimates, the majority were methodologically appropriate for describing care pathway structures, components, workforce roles, and implementation processes.

Based on the GRADE-CERQual assessment, confidence in the main synthesized structural and organizational findings ranged from moderate to high. Higher-confidence findings related to variability in pathway scope, the centrality of care coordination, multidisciplinary working, and caregiver involvement, which were supported by coherent patterns across multiple studies and contexts. Findings related to personalized care planning, digital integration, workforce composition, and training needs were generally supported by moderate-confidence evidence because of greater heterogeneity in reporting and implementation detail. Supporting CERQual judgments for each synthesized finding are presented in Supplementary File S2.

### 3.4 Evidence-based dementia care pathways identified in Europe

The studies examining evidence-based dementia care pathways in Europe showed substantial variability in scope, content, and implementation across countries and healthcare systems. On the included studies a subset described complete care pathways, while the majority focused on specific phases or modules of the pathway, such as diagnostic procedures, care planning, or follow-up. Detailed numerical data and study-level references are presented in Table 3.

**TABLE 3:** Evidence-based dementia care pathways in Europe.

Domain/element	No. of studies (*N* = 81)	Study
Pathway scope		
Complete pathway	23	(14, 36, 55, 67–69, 81, 83, 88, 93, 95, 96, 98, 99, 102, 107–109, 112, 116, 117, 119, 121)
Partial module/phase	58	(12, 47–54, 56–66, 70–80, 82, 84–92, 94, 97, 100, 101, 103–106, 110, 111, 113–115, 118, 120, 122, 123)
Phases addressed		
Referral	36	(12, 36, 47, 48, 51–53, 55, 57, 60, 64, 67, 69, 76, 77, 81, 83–87, 91, 93, 96–98, 100, 101, 105, 107, 116, 119–123)
Diagnosis assessment/disclosure	46	(12, 14, 36, 47–49, 51–56, 60, 64, 67–70, 73, 75, 77, 79, 81–88, 93–99, 101, 104, 107, 115–117, 119, 121)
Care management/coordination	68	(12, 14, 36, 47–49, 51–61, 65–71, 73–75, 77–88, 90, 91, 93–101, 103–107, 110, 111, 113–123)
End-of-life care	12	(12, 54, 55, 65, 85, 88, 99, 100, 103, 106, 117, 120)
Core components		
Case management	26	(47, 53–55, 57, 61, 73–75, 77, 78, 80–83, 91, 98, 105–109, 112, 117, 118, 123)
Patient and caregiver support	60	(14, 36, 47–53, 55–61, 65–71, 73–75, 77, 78, 80, 82–86, 88–93, 96–100, 103–107, 111, 113–117, 120–123)
Multidisciplinary teams coordination	58	(12, 14, 36, 47–49, 51, 52, 55, 56, 58–61, 65–70, 73, 77, 78, 81, 83–89, 91, 93–99, 101–111, 113–116, 119, 120, 122, 123)
Coordinated follow-up and continuity	52	(36, 47, 51–53, 55, 57–63, 65, 68–71, 73–75, 78–80, 82–84, 86–91, 93, 95–100, 104, 105, 107, 110, 111, 114–117, 119, 121, 123)
Personalized care planning	47	(12, 36, 47, 48, 51, 52, 55, 57, 60–63, 65, 66, 69, 71, 73–75, 78, 80, 82–86, 88, 90, 91, 96–100, 103–107, 111, 113–117, 120, 123)
Treatment decision	31	(36, 49, 52, 55, 59–61, 65, 68, 70, 73, 81, 83, 85–88, 93–95, 97–99, 107, 110, 115–117, 120, 121)
Structured referral systems	19	(12, 47, 51–53, 55, 57, 69, 81, 83, 84, 87, 91, 98, 102, 107, 109, 116, 118)
Volunteering services	3	(91, 100, 118)
Non-pharmacological interventions	36	(36, 51–53, 55, 60–63, 65, 66, 71, 73, 80, 83, 88, 92, 93, 96, 97, 99, 103–106, 108, 109, 113–120, 123)
Training/educational programs	42	(36, 50–53, 55, 57, 60, 62, 63, 66, 67, 69–71, 77, 80, 82–84, 87–94, 99, 100, 102–105, 109, 113–115, 117, 118, 120, 121)
Advance care planning/palliative care	11	(12, 54, 55, 65, 85, 88, 99, 100, 103, 106, 120)
Assistive technology (telehealth, teleconsultations, remote monitoring, E-learning, e-coaching, electronic care plans, clinical decision support system, intervention-management system, mobile applications)	26	(36, 50, 58, 59, 61, 64, 70, 74–76, 78, 80, 81, 83, 87, 88, 90–92, 94, 100, 107, 109, 113, 114, 116)
Workforce roles		
Caregivers involvement	57	(12, 14, 36, 48, 50–53, 55, 57–61, 63, 65–67, 69–71, 74, 75, 77–80, 82–93, 96–100, 102, 104–106, 108, 114–117, 119–122)
Case managers/coordinators	25	(54, 55, 57, 61, 73–75, 77, 80–84, 91, 100, 103, 105–109, 112, 118, 122, 123)
Primary care physicians/GPs	40	(12, 14, 36, 47, 51, 52, 56, 64, 66–70, 74–76, 78–84, 86–89, 95, 99–101, 105, 107, 108, 110, 113, 116, 119, 121, 122)
Neurologists	26	(12, 36, 55, 58, 59, 65, 70, 73, 81, 85, 87, 88, 93–97, 99, 101, 104, 107, 110, 116, 119, 122, 123)
Geriatricians	9	(12, 14, 36, 87, 89, 97, 98, 108, 119)
Psychiatrists	5	(49, 89, 97, 119, 122)
Specialist nurses (e.g., Admiral, Parkinson, dementia)	40	(12, 14, 47, 54, 57–63, 65, 66, 69, 73, 77, 80, 81, 83, 84, 87, 90, 93, 98, 99, 103–107, 111–117, 119, 120, 123)
Psychologists/neuropsychologists/ social care providers	19	(36, 49, 67, 69–71, 87, 90, 93, 94, 96, 97, 99, 104, 107, 113, 116, 119, 121)
Occupational therapists	11	(49, 51, 52, 55, 87, 89, 96, 99, 100, 107, 123)
Palliative care specialists	3	(65, 117, 120)
Memory clinic specialists	7	(36, 67, 69, 70, 93, 94, 119)
Non-clinical facilitators/volunteers	5	(53, 91, 100, 115, 118)
Training needs		
Clinical leadership/care coordination/dementia-specific care	40	(12, 14, 36, 47, 51–54, 57, 60, 61, 66–70, 80–84, 87, 88, 93, 98, 99, 102–105, 107, 112, 113, 115–120, 123)
Intervention delivery and patient/caregiver coaching	33	(47, 50–53, 55, 57, 60–63, 71, 79–85, 88–91, 97, 99, 103–105, 113, 114, 117, 118, 120)
Cultural competence and structured support	12	(12, 47, 62, 66, 77, 82, 103, 105, 112, 114, 119, 121)
Digital tools/telehealth implementation	27	(36, 50, 56, 58, 59, 61, 62, 64, 70, 74–76, 78–81, 83, 88, 90–92, 94, 107, 113, 114, 116)
Biomarkers, imaging, infusion delivery	6	(36, 70, 87, 93, 94, 102)
Ethical communication/end-of-life decision-making	3	(99, 117, 120)
Barriers to implementation		
Specialist shortages/limited access	20	(12, 36, 47, 55, 57, 68, 81, 87, 88, 95, 99, 102, 105, 106, 110, 116, 117, 119–121)
Fragmented services/poor integration	46	(12, 14, 36, 47–49, 51–59, 66–69, 74, 77, 79, 82, 85–87, 89, 94, 95, 99, 100, 102–107, 110, 111, 115–117, 119, 121–123)
Training gaps	38	(12, 36, 47, 51, 52, 57–59, 62, 63, 66–71, 79, 82, 83, 85, 87, 88, 93–95, 99, 102, 103, 105, 106, 111–117, 119, 121)
Digital literacy/technology access	19	(50, 56, 58, 61, 62, 64, 66, 70, 74, 78–80, 87, 88, 90, 92, 94, 113, 114)
Financial and infrastructure limitations	32	(12, 47, 51, 55–57, 59, 63, 66, 70, 82, 87–89, 95, 99, 102–106, 110, 112–114, 116–119, 121, 122)
Unequal access/ cultural, socioeconomic, geographic disparities	27	(12, 36, 47, 50, 52, 56, 57, 59, 65, 68, 77, 87, 88, 90, 95, 99, 102, 105–107, 111, 112, 116, 117, 119, 121, 122)
Heterogeneous implementation	55	(12, 36, 47, 48, 51–53, 55, 57–65, 70, 73, 74, 77, 79, 80, 82, 83, 85–90, 94, 95, 97–100, 102–107, 110–117, 119, 121–123)
Facilitators to implementation		
Specialized training/care coordinators	50	(12, 36, 47, 48, 51–55, 57, 60–63, 66, 69–71, 73, 74, 77, 80, 82, 83, 85, 87–91, 93, 94, 97–100, 102, 103, 105–107, 111–116, 120, 122, 123)
Multidisciplinary teamwork/collaboration	50	(12, 36, 47–49, 51, 52, 55–61, 65–69, 73, 77, 78, 83, 85–88, 91, 93, 94, 96–99, 101–103, 105–107, 110–115, 117, 119, 122, 123)
Telehealth/digital tools	26	(36, 50, 56, 58, 59, 61, 62, 64, 70, 74–76, 78–80, 83, 88, 90–92, 94, 100, 107, 113, 114, 116)
Patient-centered/supportive communication/home-based interventions	54	(12, 36, 47–53, 55–69, 71, 73–75, 77, 79, 80, 82, 83, 85, 88–92, 96, 98–100, 103, 105–107, 111, 112, 114, 115, 117, 121, 123)
Clear pathway communication/improved care coordination structures/better integration across care settings	15	(14, 36, 67–71, 83, 93, 94, 99, 115, 117, 119, 121)
Co-design with patients and caregivers/stakeholder involvement	27	(14, 36, 48, 51, 55–58, 60, 61, 66, 77, 81, 82, 86–88, 93, 102, 104, 107, 112, 113, 115–118)
Leadership/local policy support	24	(12, 36, 51, 52, 55, 60, 61, 66, 69, 70, 80, 82, 83, 86–88, 93, 98, 99, 102, 103, 105, 112, 117)

Across the different phases of care, the most frequently addressed component was care management/coordination, followed by diagnostic assessment and disclosure and referral processes. In contrast, end-of-life care was notably underrepresented, indicating that later stages of the care trajectory were less commonly reported in the literature.

Analysis of pathway components showed strong emphasis on multidisciplinary team involvement and coordinated follow-up as distinct but frequently co-reported features of care pathways. Multidisciplinary teams were frequently described, reflecting the involvement of multiple professional groups in care delivery, while coordinated follow-up and monitoring referred to mechanisms supporting continuity of care over time. Treatment decision-making and personalized care planning were also commonly reported components. Case management was present in a substantial subset of studies, indicating that it was frequently described as part of care pathway design; however, the included studies do not allow conclusions about its effectiveness or causal impact on outcomes. Caregiver support was widely integrated. Non-pharmacological interventions and training or educational programs for staff were also common components, whereas volunteering services and advance care planning or palliative care were much less frequently addressed. The incorporation of assistive and digital technologies, such as telehealth, e-coaching, and electronic care plans, was reported in a subset of studies, primarily to support communication, monitoring, and follow-up within care pathways. However, the included studies did not allow for assessment of temporal trends or increases over time in the adoption of these technologies.

Regarding workforce roles, caregiver involvement emerged as one of the most frequently reported elements. Primary care physicians and specialist nurses were consistently involved in pathway delivery, indicating that these roles were commonly represented in the included studies rather than demonstrating their relative importance in practice. Neurologists, case managers, and psychologists or neuropsychologists were also widely represented. In contrast, roles such as geriatricians, palliative care specialists, and non-clinical facilitators or volunteers were less prominent.

The studies also highlighted substantial training needs across European dementia pathways. Training on clinical leadership, care coordination, and dementia-specific competencies appeared frequently, while intervention delivery and coaching for patients and caregivers were also commonly addressed. Digital literacy and telehealth implementation were increasingly reported. Cultural competence and structured support, along with training on biomarkers, imaging, or infusion therapies, were less frequently documented. Ethical communication, particularly in relation to end-of-life decisions, appeared in only a small number of studies, indicating that this aspect was less frequently reported in the literature; in these studies, it was primarily discussed in relation to end-of-life decision-making, although the limited number of studies does not allow conclusions about the extent of professional preparation in practice.

Several barriers to implementation emerged recurrently across studies. Fragmented services and poor integration were the most frequently reported obstacles, followed by training gaps and financial or infrastructural limitations. Specialist shortages and limited access to services, digital literacy barriers, and socioeconomic, cultural, or geographic disparities further constrained the implementation and scalability of dementia pathways. These barriers were reported within individual studies and were typically linked to the implementation of specific interventions or pathway components, rather than consistently evaluated at the level of whole care pathways. Heterogeneous implementation practices highlighted persistent inconsistencies both within and across countries.

Conversely, several facilitators to implementation were identified, including specialized training and care coordination roles, multidisciplinary teamwork and collaboration, and the adoption of telehealth and digital tools. Patient-centered approaches, supportive communication, and home-based interventions were also widely acknowledged as enablers. Clear communication of pathway components and improved integration structures were cited in several studies, whereas co-design with patients, caregivers, and stakeholders and strong leadership or local policy support were also reported. These facilitators were described in relation to implementation within individual study contexts, although their effects on the adoption and sustainability of whole care pathways were not consistently evaluated, and findings relate primarily to implementation within specific health systems rather than across countries.

For detailed numerical data, study references, and extraction matrices (see Table 3 and Supplementary File S3).

### 3.5. Measurement tools, outcomes, and quality indicators in European dementia care pathways

The studies included in this review employed a wide selection of measurement tools, outcome metrics, and quality indicators, reflecting the complexity of comparing dementia care pathways across Europe. Assessments covered cognitive, functional, behavioral, caregiver-related, service-level, and feasibility domains, providing a multidimensional understanding of pathway performance.

Within the category of measurement tools, cognitive assessments were frequently applied, using established instruments such as the MMSE, MoCA, ACE-III, CERAD-NB, DemTect, and digital tools like cCOG or ICA. Diagnostic tools, including ICD-10 coding frameworks and structured screening measures such as GDS-7, were also reported. Functional and behavioral outcomes were extensively assessed, drawing on ADL/IADL scales, the NPI, DAD, UPDRS, PD-NMS, ALSFRS-R, HADS, CMAI, and wearable-derived metrics. Measures of quality of life were equally prominent, using QoL-AD, DEMQOL, EQ-5D, PDQ-39, RAND-36, and SF-12. Caregiver outcomes were captured through burden and capability scales, as well as measures of self-efficacy, mood, anxiety, or depression.

Although a small number of studies referenced biomarkers or neuroimaging tools, these appeared primarily as recommended or proposed elements for strengthening diagnostic precision in future pathway designs, rather than as measurement instruments actively used to evaluate care pathways.

Additional measures included care outcomes such as institutionalization or mortality, service utilization metrics, and indicators of staff competence, assessed through training records, guideline adherence, and competency evaluations. Feasibility and usability assessments relied on questionnaires and interviews capturing user and provider perceptions, while fidelity measures were used to assess adherence to intervention protocols.

Across the included studies, a comprehensive range of outcomes was reported. Patient-level outcomes included improvements in diagnostic accuracy and timeliness, symptom management and treatment adherence, and quality of life or well-being. Reductions in institutionalization or mortality were less commonly documented. Caregiver outcomes frequently demonstrated increased satisfaction or self-efficacy, improvements in caregiver quality of life, and reductions in burden, depression, or anxiety. Staff outcomes indicated increased competency, confidence, adherence to recommended practices, and enhanced role clarity. Service-level outcomes reflected improvements in early detection, access, referral accuracy, coordination, and continuity of care. Economic outcomes were less commonly reported, although some studies documented improved efficiency or reduced costs. A small number of studies addressed palliative or end-of-life outcomes.

A diverse set of quality indicators was also used to evaluate pathway performance. Patient and caregiver satisfaction indicators were widely reported, focusing on communication quality, timing and clarity of diagnosis, acceptability, and involvement in decisions. Educational indicators assessed training quality and staff competence. Access and equity indicators examined wait times, appropriateness of referrals, and disparities in service access. Clinical indicators integrated outcomes such as quality of life, neuropsychiatric symptoms, caregiver burden, diagnostic accuracy, and hospitalization rates. Finally, economic indicators considered service costs, cost-effectiveness, and QALYs, contributing insight into financial and system-level impacts of pathway innovations. Detailed study-level data and full reference lists are provided in Table 4 and Supplementary File S3.

**TABLE 4:** Measurement Tools, outcomes and quality indicators.

Domain/element	Subcategory/feature	No. of studies (*N* = 81)	Study
Measurement tools			
Cognitive assessments	MMSE/MoCA/DemTect/ACE-III/CERAD-NB/cCOG/PANDA/ICA	19	(36, 52, 60, 64, 70, 73, 75, 76, 78–80, 83, 84, 93, 94, 96, 97, 102, 108)
Diagnostic tools	ICD-10 codes (F00–F03, F05.1, G30)/GDS-7	7	(52, 55, 68, 78, 95, 110, 120)
Patient outcomes Functional/behavioral	ADL/IADL/Bayer ADL/FAQ/DAD/AGGIR/NPI/agitation/ apathy/UPDRS/PD-NMS/BDI-II/ALSFRS-R/MDS-UPDRS/accelerometer/NMSQ/PDSS-2/HADS/CANE/CMAI/GDS-15/IPOS/wearable metrics	29	(51–53, 55, 59–63, 65, 73, 75, 76, 78–81, 83, 84, 88, 91, 96, 97, 105, 106, 110, 115, 116, 118)
Quality of life	QoL-AD/DEMQOL/EQ-5D/PDQ-39/RAND-36/ALSAQ-40/WHODAS/SF-12/SF-6D	29	(50, 52, 53, 57–63, 65, 73, 76, 79, 80, 83, 89–92, 105, 108, 110, 111, 115, 116, 118, 121, 123)
Caregiver outcomes	Zarit/BICB/ASCOT	11	(53, 57, 59, 60, 65, 79, 80, 83, 100, 105, 108)
Self-efficacy/anxiety/depression (PHQ-9)	14	(50–53, 57, 60, 77, 79, 90–92, 100, 105, 108)
Biomarkers	CSF/PET/MRI/plasma/Aβ42/40	8	(36, 55, 87, 93, 94, 102, 107, 109)
Care outcomes	Institutionalization/Mortality (nursing home logs, death registries)	10	(12, 68, 83, 89, 95–97, 106, 112, 121)
Service utilization	Care usage/continuity/costs/referral accuracy/readmissions Medication use/PIMs (patient records, admin databases)	29	(12, 47, 50, 52, 59, 61, 63–65, 68, 77, 80, 83, 85, 87, 89, 94, 95, 98, 100, 101, 105, 106, 112, 115, 116, 119, 121, 122)
Staff competence	Competence/training/adherence to guidelines (competency assessments, training records)	26	(12, 47, 51, 52, 55, 62, 66, 79, 82–85, 87, 90, 91, 94, 95, 102–104, 110, 113, 115, 119–122)
Feasibility/usability	User perceptions (questionnaires/interviews)	31	(56–62, 65, 66, 74, 77–79, 82–84, 86, 88, 94, 96, 102–105, 111–116, 118)
Fidelity metrics	IMS-DCM concordance	7	(63, 65, 74, 78, 83, 103, 105)
Outcomes			
Patient outcomes	Diagnostic accuracy/timeliness (improvement)	18	(12, 36, 52, 64, 70, 76, 84, 87, 88, 93–95, 99, 101, 102, 107, 109, 116)
	Symptom management/treatment adherence/(improvement)	24	(36, 51, 52, 58, 59, 61–63, 65, 73, 80, 81, 83, 88, 93, 95, 96, 99, 105, 106, 108, 110, 112, 115, 116)
	Quality of life/well-being (improvement)	28	(14, 51, 52, 58, 59, 61–63, 65, 73, 77, 79–81, 83, 88–90, 99, 108, 110–112, 114–116, 118, 123)
	Institutionalization/mortality (reduction/delayed)	9	(12, 68, 83, 89, 96, 97, 99, 106, 112)
Caregiver outcomes	Satisfaction/self-efficacy (improvement)	24	(48, 50–53, 57–59, 67, 77, 84, 89–93, 96, 100, 108, 112, 114, 115, 118, 122)
	Quality of life/well-being (improvement)	12	(14, 50–53, 57, 65, 77, 89–91, 108)
	Burden/depression/anxiety (reduction)	17	(50–53, 57, 59, 60, 65, 77, 79, 80, 83, 99, 100, 105, 108, 121)
Staff competence	Adherence/training level/knowledge/skills/self-efficacy/attitudes/role clarity (increase)	26	(12, 47, 51, 52, 62, 66, 71, 79, 82, 84, 85, 87, 90, 91, 93, 102–104, 109, 110, 113, 115, 117, 119, 121, 122)
Service outcomes	Early detection/access/referral accuracy/continuity/coordination (improvement/reduction where appropriate)	47	(12, 36, 47–50, 52, 56, 59, 61, 64, 66–68, 70, 73, 74, 77, 82–85, 87–89, 92–95, 98–102, 105–110, 112, 114–116, 118, 119, 122)
	Costs/cost-effectiveness (reduction/improved efficiency)	9	(50, 63, 65, 80, 89, 95, 101, 108, 115)
Palliative/end-of-life	ACP/comfort (improvement)	4	(54, 99, 106, 112)
Quality Indicators			
Patient and caregiver satisfaction	Communication/timing of diagnosis/acceptability/perceived usefulness/involvement	39	(12, 14, 47–49, 51, 52, 56, 57, 59, 61, 62, 65, 67, 69, 73, 74, 77, 83–85, 88–93, 95–97, 100, 102, 104, 105, 107, 109, 112, 114, 115)
Educational outcome	Competence/training quality/satisfaction	9	(83, 84, 93, 103, 113, 115, 118, 119, 121)
Access and equity	Wait times/equity/referral appropriateness	35	(12, 14, 36, 47, 48, 52, 54–57, 59, 64, 67–69, 77, 81–84, 87, 88, 90, 95, 99, 102, 107, 109, 112, 114, 116, 118, 119, 121, 122)
Clinical outcomes	QoL/NPI/caregiver burden/diagnostic accuracy/hospitalization rates	47	(12, 36, 47, 50–53, 55, 59, 61–66, 76, 79, 80, 83–85, 87–91, 94, 95, 97–102, 104–111, 115–118, 123)
Economic indicators	Service costs/cost-effectiveness/QALY	18	(47, 50, 57, 63, 65, 80, 81, 88, 89, 92, 95, 101, 102, 108, 112, 115, 116, 122)

MMSE, Mini-Mental State Examination; MoCA, Montreal Cognitive Assessment; DemTect, Dementia Detection Test; ACE-III, Addenbrooke’s Cognitive Examination–III; CERAD-NB, Consortium to Establish a Registry for Alzheimer’s Disease Neuropsychological Battery; cCOG, computerized Cognitive Test Battery; PANDA, Parkinson Neuropsychometric Dementia Assessment; ICA, Integrated Cognitive Assessment; F00–F03, F05.1, G30, ICD-10 diagnostic codes; ADL, Activities of Daily Living; IADL, Instrumental Activities of Daily Living; Bayer-ADL, Bayer Activities of Daily Living Scale; FAQ, Functional Activities Questionnaire; DAD, Disability Assessment for Dementia; AGGIR, AGGIR Functional Autonomy Scale; NPI, Neuropsychiatric Inventory; CMAI, Cohen-Mansfield Agitation Inventory; UPDRS III; MDS-UPDRS II–III, Unified Parkinson’s Disease Rating Scale; PD-NMS; NMSQ, Parkinson’s Non-Motor Symptoms Questionnaire; PDSS-2, Parkinson Disease Sleep Scale–2; BDI-II, Beck Depression Inventory–II; HADS, Hospital Anxiety and Depression Scale; CANE, Carers’ Needs Assessment for the Elderly; ALSFRS-R, ALS Functional Rating Scale–Revised; GDS-15, Geriatric Depression Scale–15; IPOS Neuro-S8, Integrated Palliative Care Outcome Scale for Neurological Conditions; QoL-AD, wearable-sensor physiological metrics; Quality of Life in Alzheimer’s Disease; DEMQOL, Dementia Quality of Life Questionnaire; EQ-5D; EQ-5D-5L, EuroQol instruments; PDQ-39; RAND-36, Parkinson’s Disease Questionnaire; ALSAQ-40, ALS Assessment Questionnaire–40; WHODAS, World Health Organization Disability Assessment Schedule; SF-12; SF-6D, Short-Form Health Surveys; ZBI, Zarit Burden Interview; BICB, Berlin Inventory of Caregiver Burden; ASCOT, PHQ-9, Adult Social Care Outcomes Toolkit; Patient Health Questionnaire–9; SSCQ, Short Sense of Competence Questionnaire; CSF, cerebrospinal fluid biomarkers; PET, positron emission tomography for amyloid and tau; MRI, magnetic resonance imaging; p-tau, plasma phosphorylated tau; Aβ42/40, amyloid-beta 42/40 ratio; institutionalization and mortality records; care utilization metrics; RUD-lite, Resource Utilization in Dementia instrument; QALYs, Quality-Adjusted Life Years; ICERs, Incremental Cost-Effectiveness Ratios; SCIDS, Sense of Competence in Dementia Care Staff Scale; POPAC-R, Person-Centered Care of Older People with Cognitive Impairment in Acute Care Scale–Revised; AIM, IAM, FIM, Acceptability, Appropriateness, and Feasibility of Intervention Measures; IMS–DCM, Intervention-Management System for Dementia Care Mapping; PIE, Person–Interaction–Environment tool; ECA, Embodied Conversational Agents; IoT, Internet of Things technologies; mHealth, and mobile health tools.

### 3.6 Conceptual synthesis of evidence-based dementia and neurodegenerative disorders care pathways

To support interpretation of the heterogeneous evidence identified in this review, we developed an evidence-informed conceptual synthesis that integrates the most consistently reported pathway elements, key domains of care delivery, and cross-cutting principles shaping pathway performance across European contexts (Figure 2). The approach used to develop this synthesis is described in the Methods section. Briefly, the model was generated through an iterative process involving (i) identification of recurrent components across studies, (ii) grouping of these components into higher-order domains based on functional similarity, and (iii) synthesis of cross-cutting principles influencing pathway implementation. This process was conducted by the review team and refined through discussion to ensure conceptual coherence and consistency with the extracted data. The model does not represent an idealized or prescriptive pathway, but an analytical synthesis grounded in the frequency, function, and variability of components reported across the included studies.

#### 3.6.1 Core pathway functions

For the purposes of this review, core pathway functions refer to the central organizational mechanisms that support the coordination, delivery, and continuity of care across pathway stages and service settings. These functions were consistently identified across the included studies and underpin the broader domains of care delivery represented in the conceptual synthesis. Across the evidence base, several elements emerged as core organizing functions of post-diagnostic dementia and neurodegenerative care. Care coordination and continuity were consistently described as enabling navigation across services, structured communication, and ongoing follow-up over time. These functions were closely associated with case management, multidisciplinary teams, and structured follow-up, which together support continuity of care across settings and stages of the disease trajectory.

#### 3.6.2 Domains of care delivery

Surrounding these core functions, the review identified key domains of care delivery, corresponding to those presented in Figure 2: diagnostic assessment and disclosure; caregiver involvement; workforce capacity and training; digital and assistive technologies; care coordination and continuity; and long-term and end-of-life care. Diagnostic assessment and disclosure were commonly well established, reflecting the prominence of clinical entry points into care pathways. Caregiver involvement emerged as a widely integrated domain, with caregivers frequently positioned as active participants in care processes through psychoeducation and shared decision-making. Workforce capacity and training were identified as enabling conditions across studies, with recurrent gaps in dementia-specific competencies, coordination skills and digital literacy. Digital and assistive technologies were incorporated in a subset of pathways, primarily to support communication, monitoring, and follow-up, although their adoption remained uneven and often limited to specific functions. Across studies, digital technologies most commonly functioned as enabling tools that supported care coordination, communication, monitoring, and service accessibility rather than as stand-alone pathway components. Their contribution was therefore conceptualized as a cross-cutting facilitator of pathway implementation, despite substantial variation in adoption and integration across settings. Care coordination and continuity extended beyond core functions into service-level organization, where coordination between primary care, specialist services, and social care remained inconsistently implemented, with fragmentation repeatedly reported. Long-term and end-of-life care, including advance care planning or palliative care, were least frequently addressed, indicating persistent gaps in pathway completeness across later disease stages.

#### 3.6.3 Cross-cutting principles

In addition to these domains, several cross-cutting principles shaped pathway quality and feasibility across contexts. Equity and access concerns were reflected in geographic, socioeconomic, and cultural disparities in access to diagnosis and specialized services. Person and caregiver-centered care consistently emerged as facilitators of engagement and acceptability. Quality indicators and monitoring practices were highly heterogeneous, with frequent use of clinical and experience-based measures but limited standardization across studies. Feasibility and sustainability were influenced by workforce capacity, resource availability, organizational support, and digital infrastructure, shaping the extent to which pathways could be implemented and maintained in routine care.

Overall, this conceptual synthesis provides a structured analytical approach for interpreting the diversity of European dementia and neurodegenerative care pathways identified in the literature. By distinguishing core functions, domains of care delivery, and cross-cutting principles aligned with Figure 2, it supports comparison across heterogeneous models and highlights priorities for more coherent, longitudinal and evaluable pathway design.

## 4 Discussion

### 4.1 Summary of main findings

This systematic review provides a detailed synthesis of how dementia and neurodegenerative disorder care pathways are structured, implemented, and evaluated across Europe, addressing long-standing fragmentation and the absence of integrated, longitudinal pathway models identified in previous studies (11–13). Drawing on 81 studies from multiple European contexts, it offers both a descriptive mapping of pathway phases, components, outcomes, and implementation factors, and an integrative conceptual interpretation of how these elements function together in practice. The included pathways were highly heterogeneous in format, scope, and intended users, confirming and extending observations from previous reviews and reflecting differences in national service organization and in the objectives pursued (e.g., standardizing diagnosis, improving coordination, strengthening post-diagnostic support) (14, 15). Importantly, this heterogeneity is itself informative: it highlights the diversity of approaches across Europe and underscores the diversity of approaches shaping dementia care.

Importantly, the findings of this review should be interpreted in relation to the published evidence base rather than as a direct representation of care pathway implementation across Europe. The included studies reflect specific research contexts, priorities, and study designs, which may not fully capture routine clinical practice or system-level organization. As such, the patterns identified should be understood as indicative of how dementia care pathways are conceptualized and studied, rather than as a comprehensive account of how they are consistently implemented across health systems.

Rather than reiterating descriptive findings, this section focuses on interpreting these patterns in relation to existing literature and their implications for pathway design and evaluation. Across the included studies, care pathways were predominantly oriented toward early-stage processes, particularly diagnostic assessment, communication of the diagnosis, and initial post-diagnostic support, while later-stage elements, such as long-term management, palliative care, and transitions to end-of-life care, were less frequently reported. These patterns, as consistently described in the literature, empirically confirm observations from previous studies suggesting that pathways have primarily evolved to standardize diagnostic and early management practices (12, 14). Given the progressive and long-term nature of neurodegenerative conditions, the limited formalization of later stages in the published evidence base may constrain continuity, anticipatory planning, and the delivery of person-centered care over time, particularly as individuals progress into more complex and resource-intensive stages of the disease trajectory (12).

Despite this variation, the evidence suggests a consistent functional core within the published literature. Care coordination, multidisciplinary teamwork, case management, and structured follow-up emerge as central organizing functions that are recurrently reported across studies rather than consistently evaluated in real-world implementation.. This interpretation aligns with the integrated dementia care literature, which describes comparable organizing principles for effective integration (23). Around this core, additional domains, such as diagnostic processes, caregiver support, workforce development, digital enablement, and quality measurement, were incorporated with varying degrees of formalization across studies.

The review highlights a set of transversal enablers that shape pathway coherence and feasibility. First, care coordination and care management were consistently described as mechanisms supporting transitions between services and continuity of care. However, their implementation remains uneven as reflected in the variability reported across studies, rather than systematically evaluated across health systems. This interpretation is consistent with previous research indicating that organizational enablers are central to integrated dementia care (24, 25).

Second, workforce training and capacity building were frequently identified as prerequisites for pathway fidelity and sustainability, reflecting long-standing recognition of the specialized competencies, coordination skills, and role clarity required in dementia care (2, 26, 27). Persistent training gaps across clinical, coordination, and digital competencies were repeatedly reported in the literature, suggesting that implementation remains challenging in routine practice, with implications for the long-term sustainability (28–30).

Third, caregiver involvement emerged as a defining cross-cutting feature across pathways, with many models positioning caregivers as co-producers of care. While this aligns with existing literature suggesting that caregiver participation may support continuity and outcomes (31–33), it also highlights a potential structural vulnerability: pathways that rely heavily on informal care may be sensitive to caregiver burden and resource constraints. These findings underscore the importance of integrating caregiver support as a core design element, although the extent to which this is achieved in practice remains unclear from the available evidence.

Several cross-cutting principles further shape implementation and evaluation. Interpretation of measurement practices should be understood in relation to what is reported in the literature rather than reflecting a standardized or consistently applied evaluation framework across European systems. The wide range of measurement tools, outcomes, and quality indicators identified reflects increasing methodological sophistication and a shift toward multidimensional evaluation. However, substantial heterogeneity in how outcomes are defined and operationalized limits comparability across studies. Consistent with previous reviews in dementia research, the absence of standardized outcome and indicator sets limits cross-study comparability and constrains the development of cohesive European evaluation and benchmarking frameworks, with implications for scaling and policy learning (13, 34, 35). While biomarkers and neuroimaging were occasionally referenced, they were rarely integrated into pathway evaluation, highlighting a gap between emerging clinical innovations and existing evaluation frameworks (36).

Feasibility and sustainability also emerged as critical cross-cutting considerations. Across the included studies, feasibility was frequently assessed through usability, acceptability, and implementation-related outcomes, while sustainability was reflected in recurring concerns related to workforce capacity, resources, and organizational support. The predominance of feasibility-oriented designs suggests that many pathways are evaluated under constrained real-world conditions, consistent with implementation literature indicating that effectiveness depends on whether models can be realistically delivered and maintained over time (37, 38).

Digital technologies represent another cross-cutting element whose integration remains uneven. Telehealth, electronic care plans, and remote monitoring tools were increasingly described, but their use was largely confined to specific functions rather than embedded across entire pathways. However, the available evidence does not allow conclusions about system-level digital transformation or trends over time across European health systems. Adoption appeared highly context-dependent, shaped by infrastructure, digital literacy, and organizational readiness (39). Nevertheless, the findings suggest that digital technologies function not merely as discrete pathway components but as transversal enablers that support coordination, communication, monitoring, information exchange, and continuity of care across multiple pathway domains. Their contribution therefore extends beyond individual interventions and may influence the integration and performance of care pathways as a whole, although the degree of integration remains highly variable across settings. Person- and caregiver-centeredness further emerged as an important transversal principle influencing acceptability and engagement. These approaches were frequently operationalized through communication, shared decision-making, and tailored care. However, as reflected in the literature, they were often implemented through discrete components rather than embedded across entire care pathways (40–42). Equity and access also emerged as critical dimensions. Socioeconomic, geographic, cultural, and linguistic disparities were frequently reported as barriers to access and continuity of care. These findings reflect reported challenges in the literature and should not be interpreted as a comprehensive assessment of real-world service provision across all European contexts (43).

The conceptual synthesis developed in this review is broadly consistent with priorities identified in international dementia policy frameworks, including the World Health Organization Global Action Plan on the Public Health Response to Dementia 2017–2025 (2), the WHO Global Status Report on the Public Health Response to Dementia (44), OECD policy reports (17), and Alzheimer’s Disease International initiatives (3, 11). Common areas of convergence include the emphasis on integrated and person-centered care, workforce development, caregiver support, care coordination, equity of access, and the use of digital technologies to strengthen service delivery. However, the present synthesis extends these policy-oriented frameworks by identifying how such priorities are operationalized within published care pathway models and by highlighting persistent gaps in implementation, particularly in relation to long-term care, palliative care, and pathway evaluation.

The findings also align with comparative analyses of national dementia strategies and policy approaches (3, 45), which have consistently highlighted challenges related to implementation, coordination across sectors, workforce capacity, and monitoring mechanisms. While many national strategies articulate similar objectives, evidence from the reviewed literature suggests substantial variability in how these priorities are translated into operational care pathways. This reinforces the importance of implementation-focused approaches that move beyond policy commitments toward the development of sustainable and evaluable service models.

Based on patterns identified across the reviewed literature, these findings suggest a set of priorities for strengthening dementia and neurodegenerative care pathways. These priorities are directly derived from the recurrent patterns and gaps identified across the included studies, particularly the limited representation of later-stage care, variability in coordination mechanisms, and heterogeneity in evaluation practices. These include (i) shifting toward integrated, longitudinal pathways; (ii) reinforcing coordination and workforce capacity; (iii) embedding long-term and end-of-life care; (iv) improving referral and access mechanisms; (v) integrating person-centered and equity approaches; and (vi) developing more consistent evaluation frameworks.

In line with European calls for more integrated systems (2, 7, 11, 46), the conceptual synthesis developed in this review (Figure 2) provides a structured framework to support interpretation, comparison, and future pathway development. The model is derived from the published evidence base and should therefore be understood as reflecting how care pathways are conceptualized and reported in the literature, rather than representing their uniform implementation across European health systems.

### 4.2 Limitations

Interpretation of the findings is limited by heterogeneity in study designs, outcome measures, and reporting practices, as well as by the predominance of evidence from early disease stages and a subset of European countries. Although studies addressing specific pathway phases were eligible when embedded within broader integrated models, much of the available evidence focuses on individual pathway components rather than complete longitudinal care trajectories, which may contribute to the underrepresentation of later-stage care elements in the literature. In addition, uneven reporting of digital integration, long-term outcomes, and palliative care constrained more granular comparative analyses across settings. These limitations primarily affect the depth of comparative and stage-specific analyses, rather than the identification of recurring structural and organizational patterns across pathways. Furthermore, although the review adopted a broader neurodegenerative perspective, the included evidence was predominantly focused on dementia. Consequently, the conceptual synthesis was primarily informed by dementia care pathway literature and may not fully capture disease-specific care requirements, service structures, or palliative care needs associated with other neurodegenerative disorders. This should be considered when interpreting the applicability of the framework across the wider spectrum of neurodegenerative conditions. The restriction to English-language publications may have limited the inclusion of national policy documents, implementation reports, and locally published studies from European countries where relevant evidence is commonly disseminated in national languages. As a result, some country-specific pathway initiatives, policy developments, or implementation experiences may not have been captured, potentially affecting the comprehensiveness of the review, particularly in relation to health system organization and policy implementation. In addition, limitations related to the review process include potential publication bias, variability in reporting across studies, and the restriction to studies published within the last 10 years.

## 5 Conclusion

This systematic review synthesizes a diverse and heterogeneous body of European evidence on dementia and neurodegenerative disorder care pathways, highlighting both substantial variation in pathway design and a set of recurrent functional patterns that transcend national and organizational contexts. While existing pathways differ widely in scope, format, and implementation, the findings demonstrate a consistent functional core centered on care coordination, multidisciplinary working, case management, and structured follow-up, which together enable continuity over time and across services. At the same time, the review reveals a persistent imbalance toward early-stage processes, with later-stage elements such as long-term management, advance care planning, palliative care, and end-of-life transitions remaining comparatively underdeveloped in the available evidence. This front-loaded orientation risks limiting continuity, anticipatory planning, and person-centered care throughout the full disease trajectory of progressive neurodegenerative conditions. By integrating pathway components, domains of care delivery, and transversal enablers into an evidence-informed conceptual synthesis, this review moves beyond descriptive mapping to clarify how dementia care pathways function in practice. The proposed framework does not prescribe a single optimal model, but provides a structured lens to support pathway design, implementation, and evaluation within diverse European health systems. Building on this framework, future efforts to strengthen dementia and neurodegenerative disorder care pathways should prioritize longitudinal integration, embed enabling functions and equity considerations across all stages of care, and adopt feasible, standardized approaches to outcome measurement that support learning, comparison, and sustainable system improvement.

## Footnotes

1
https://www.crd.york.ac.uk/PROSPERO/view/CRD420251020962
