# Supplementary material for: Evidence-based care pathways for people with dementia and neurodegenerative disorders in Europe: a systematic review of models, integration of digital technologies, and quality indicators
Source: Front Med (Lausanne). 2026 Jun 29;13:1774318. doi: 10.3389/fmed.2026.1774318 (PMC13357144; doi:10.3389/fmed.2026.1774318)
Supplement: Supplementary file 1 [file Data_Sheet_1.PDF]

Systematic Review Search Strategy

Review title:

Evidence-Based Care Pathways for People with Dementia and Neurodegenerative Disorders: including Models, Integration of Digital Technologies, and Quality Indicators.

The search strategy for this systematic review was developed and conducted by Dr. Maria Isabel Cardona and a colleague from the German Center for Neurodegenerative Diseases (DZNE), a researcher who assisted with double-review processes and ensuring the validity of study inclusion. Both researchers have experience in conducting systematic reviews and data extraction.

The strategy has been reviewed, piloted, and refined by the team to ensure thoroughness, consistency, and accuracy. It was updated as needed to accommodate specific database requirements and controlled vocabulary. This ensures that the strategy remains effective across multiple platforms. The following databases were searched:

- PubMed
- Embase
- CINAHL
- Cochrane Library
- Web of Science

Manual Search of Reference Lists:

The review team manually searched the reference lists of relevant studies and key articles to identify additional sources that may not have been captured through the database search.

Grey Literature:

To expand the search and capture a broader range of evidence, the review also included grey literature, such as reports, working papers, and policy documents from key organizations, including:

- European Commission
- World Health Organization (WHO)
- Alzheimer's Europe
- JADEHealth project reports (to capture data from participating countries on the status and integration of dementia care pathways).

Inclusion and Exclusion Criteria:

Inclusion Criteria:

- **Population:** People with dementia or neurodegenerative disorders; informal (e.g., family, friends) and formal (e.g., healthcare professionals, social workers) caregivers.
- **Interventions:** Evidence-based health and social care pathways, including structured clinical and service pathways.
- **Outcomes:** Quality of care, patient and caregiver outcomes, healthcare efficiency, service integration, barriers, and facilitators.
- **Study Design:** Randomized controlled trials (RCTs), systematic reviews, cohort studies, clinical guidelines, and relevant policy reports.
- **Countries**: Studies conducted in Eropean countries.
- **Timeframe:** Studies published in the last 10 years.

Exclusion Criteria:

- Studies that do not focus on dementia or neurodegenerative disorders.

- Studies that focus solely on acute care or hospital-based settings, excluding long-term care or home-based care pathways.
- Studies that do not describe or evaluate structured clinical or service pathways.
- Studies that focus exclusively on pharmacological treatments or interventions without considering care pathways.
- Studies published before the last 10 years.

Ongoing Monitoring and Alerts:

Monthly search alerts were set up in each database to ensure the inclusion of any new studies published during the review period. These alerts were monitored to ensure that the most recent and relevant studies were incorporated into the review.

**PubMed Search Strategy**	
Line	Search Terms
1	Dementia[mh] OR Alzheimer's Disease[mh] OR dementia[tiab] OR cognitive decline[tiab] OR memory loss[tiab] OR neurocognitive disorders[tiab] OR early-onset dementia[tiab] OR progressive cognitive impairment[tiab]
2	Neurodegenerative Disorders[mh] OR Parkinson's disease[tiab] OR Huntington's disease[tiab] OR Amyotrophic lateral sclerosis[tiab] OR ALS[tiab] OR neurodegenerative[tiab] OR motor neuron disease[tiab] OR Lewy body dementia[tiab] OR frontotemporal dementia[tiab]
3	**#2 OR #3**
4	Evidence-Based Care Pathways[tiab] OR clinical pathways[tiab] OR care models[tiab] OR structured care[tiab] OR healthcare pathways[tiab] OR integrated care pathways[tiab] OR evidence-based care models[tiab] OR care frameworks[tiab] OR multidisciplinary care[tiab]
5	Clinical Pathways[tiab] OR treatment pathways[tiab] OR care protocols[tiab] OR management pathways[tiab] OR diagnostic pathways[tiab] OR standard care plans[tiab]
6	Integrated Care[tiab] OR integrated healthcare[tiab] OR coordinated care[tiab] OR collaborative care[tiab] OR person-centered care[tiab] OR holistic care[tiab] OR multi-disciplinary care[tiab] OR team-based care[tiab] OR patient-centered integrated care[tiab] OR care integration[tiab]
7	Digital Health Technologies[tiab] OR telemedicine[tiab] OR telehealth[tiab] OR mobile health[tiab] OR e-health[tiab] OR digital health[tiab] OR health apps[tiab] OR remote monitoring[tiab] OR wearable health technology[tiab] OR virtual health[tiab] OR health information technology[tiab] OR digital health interventions[tiab] OR health IT solutions[tiab]
8	Health Technology Assessment[tiab] OR HTA[tiab] OR health technology evaluation[tiab] OR digital health assessment[tiab] OR health interventions[tiab] OR effectiveness of digital tools[tiab] OR health tech evaluations[tiab] OR economic evaluation[tiab] OR impact assessment[tiab] OR medical technology assessment[tiab]
9	**#5 OR #6 OR #7 OR #8 OR #9**
10	Standard Care[tiab] OR usual care[tiab] OR conventional care[tiab] OR routine care[tiab] OR current care practices[tiab] OR traditional care[tiab]
11	Traditional Care Models[tiab] OR non-integrated care[tiab] OR non-digital care[tiab] OR usual models of care[tiab] OR non-coordinated care[tiab] OR conventional treatment models[tiab]
12	**#11 OR #12**
13	Caregivers[tiab] OR family caregivers[tiab] OR informal caregivers[tiab] OR primary caregivers[tiab] OR dementia caregivers[tiab] OR unpaid caregivers[tiab] OR caregiving[tiab] OR caregiver burden[tiab] OR caregiving challenges[tiab]

14	Healthcare Professionals[tiab] OR nurses[tiab] OR doctors[tiab] OR physicians[tiab] OR social workers[tiab] OR healthcare providers[tiab] OR care managers[tiab] OR geriatric specialists[tiab] OR healthcare staff[tiab] OR dementia specialists[tiab] OR medical professionals[tiab]
15	**#14 OR #15**
16	Quality of Care[tiab] OR healthcare quality[tiab] OR patient care quality[tiab] OR healthcare services quality[tiab] OR quality indicators[tiab] OR patient satisfaction[tiab] OR care standards[tiab] OR treatment quality[tiab]
17	Patient Outcomes[tiab] OR health outcomes[tiab] OR treatment outcomes[tiab] OR patient health[tiab] OR recovery outcomes[tiab] OR morbidity[tiab] OR mortality[tiab] OR functional outcomes[tiab] OR quality of life[tiab]
18	Healthcare Efficiency[tiab] OR care efficiency[tiab] OR healthcare productivity[tiab] OR healthcare service efficiency[tiab] OR cost-effectiveness[tiab] OR service delivery efficiency[tiab] OR health system efficiency[tiab] OR care resource utilization[tiab]
19	Service Integration[tiab] OR care coordination[tiab] OR healthcare integration[tiab] OR service delivery[tiab] OR multi-level integration[tiab] OR service integration models[tiab] OR healthcare service integration[tiab] OR cross-sector integration[tiab]
20	Barriers and Facilitators[tiab] OR implementation challenges[tiab] OR barriers to care[tiab] OR obstacles to care integration[tiab] OR enablers[tiab] OR facilitators[tiab] OR implementation facilitators[tiab] OR care implementation barriers[tiab]
21	**#17 OR #18 OR #19 OR #20 OR #21**
22	RCT OR randomized controlled trial[tiab] OR cohort study[tiab] OR systematic review[tiab] OR meta-analysis[tiab] OR clinical trial[tiab] OR clinical guideline[tiab] OR policy report[tiab] OR qualitative study[tiab] OR mixed methods study[tiab]
23	**#4 AND #10 AND #13 AND #16 AND #22 AND #23**

**Cochrane Search Strategy**	
Line	Search Terms
1	Dementia OR Alzheimer's Disease OR Cognitive Decline OR Neurocognitive Disorders OR Memory Loss OR Early-Onset Dementia
2	Neurodegenerative Disorders OR Parkinson’s Disease OR Huntington’s Disease OR Amyotrophic Lateral Sclerosis (ALS) OR Motor Neuron Disease OR Lewy Body Dementia OR Frontotemporal Dementia
3	**#2 OR #3**
4	Care Pathways OR Clinical Pathways OR Care Models OR Structured Care OR Integrated Care Pathways OR Evidence-Based Care Models
5	Treatment Pathways OR Care Protocols OR Management Pathways OR Diagnostic Pathways OR Standard Care Plans
6	Integrated Care OR Coordinated Care OR Collaborative Care OR Person-Centered Care OR Holistic Care OR Multi-Disciplinary Care OR Team-Based Care
7	Digital Health Technologies OR Telemedicine OR Telehealth OR Mobile Health OR E-Health OR Health Apps OR Remote Monitoring OR Wearable Health Technology OR Virtual Health OR Health IT Solutions
8	Health Technology Assessment (HTA) OR Health Technology Evaluation OR Digital Health Assessment OR Health Interventions OR Effectiveness of Digital Tools OR Health Tech Evaluations OR Economic Evaluation
9	**#5 OR #6 OR #7 OR #8 OR #9**
10	Standard Care OR Usual Care OR Conventional Care OR Routine Care OR Current Care Practices OR Traditional Care
11	Traditional Care Models OR Non-Integrated Care OR Non-Digital Care OR Usual Models of Care OR Non-Coordinated Care OR Conventional Treatment Models

12	**#11 OR #12**
13	Caregivers OR Family Caregivers OR Informal Caregivers OR Primary Caregivers OR Dementia Caregivers OR Unpaid Caregivers OR Caregiving OR Caregiver Burden OR Caregiving Challenges
14	Healthcare Professionals OR Nurses OR Doctors OR Physicians OR Social Workers OR Healthcare Providers OR Care Managers OR Geriatric Specialists OR Dementia Specialists
15	**#14 OR #15**
16	Quality of Care OR Healthcare Quality OR Patient Care Quality OR Healthcare Services Quality OR Quality Indicators OR Patient Satisfaction OR Care Standards
17	Patient Outcomes OR Health Outcomes OR Treatment Outcomes OR Patient Health OR Recovery Outcomes OR Morbidity OR Mortality OR Functional Outcomes OR Quality of Life
18	Healthcare Efficiency OR Care Efficiency OR Healthcare Productivity OR Healthcare Service Efficiency OR Cost-Effectiveness OR Service Delivery Efficiency OR Health System Efficiency OR Care Resource Utilization
19	Service Integration OR Care Coordination OR Healthcare Integration OR Service Delivery OR Multi-Level Integration OR Healthcare Service Integration
20	Barriers and Facilitators OR Implementation Challenges OR Barriers to Care OR Obstacles to Care Integration OR Enablers OR Facilitators OR Implementation Facilitators
21	**#17 OR #18 OR #19 OR #20 OR #21**
22	RCT OR Randomized Controlled Trial OR Systematic Review OR Meta-Analysis OR Clinical Trial OR Clinical Guideline OR Policy Report OR Qualitative Study OR Mixed Methods Study
23	**#4 AND #10 AND #13 AND #16 AND #22 AND #23**

**Embase Search Strategy**	
Line	Search terms
1	dementia'/exp OR 'alzheimer disease'/exp OR 'dementia':ti,ab,kw OR 'cognitive decline':ti,ab,kw OR 'memory loss':ti,ab,kw OR 'neurocognitive disorders':ti,ab,kw OR 'early onset dementia':ti,ab,kw OR 'progressive cognitive impairment':ti,ab,kw
2	degenerative disease'/exp OR 'parkinson disease':ti,ab,kw OR 'huntington disease':ti,ab,kw OR 'amyotrophic lateral sclerosis':ti,ab,kw OR 'als':ti,ab,kw OR 'neurodegenerative':ti,ab,kw OR 'motor neuron disease':ti,ab,kw OR 'lewy body dementia':ti,ab,kw OR 'frontotemporal dementia':ti,ab,kw
3	**#2 OR #3**
4	evidence based care pathways':ti,ab,kw OR 'clinical pathways':ti,ab,kw OR 'care models':ti,ab,kw OR 'structured care':ti,ab,kw OR 'healthcare pathways':ti,ab,kw OR 'integrated care pathways':ti,ab,kw OR 'evidence based care models':ti,ab,kw OR 'care frameworks':ti,ab,kw OR 'multidisciplinary care':ti,ab,kw
5	clinical pathways':ti,ab,kw OR 'treatment pathways':ti,ab,kw OR 'care protocols':ti,ab,kw OR 'management pathways':ti,ab,kw OR 'diagnostic pathways':ti,ab,kw OR 'standard care plans':ti,ab,kw
6	integrated care':ti,ab,kw OR 'integrated healthcare':ti,ab,kw OR 'coordinated care':ti,ab,kw OR 'collaborative care':ti,ab,kw OR 'person centered care':ti,ab,kw OR 'holistic care':ti,ab,kw OR 'multi disciplinary care':ti,ab,kw OR 'team based care':ti,ab,kw OR 'patient centered integrated care':ti,ab,kw OR 'care integration':ti,ab,kw
7	digital health technologies':ti,ab,kw OR 'telemedicine':ti,ab,kw OR 'telehealth':ti,ab,kw OR 'mobile health':ti,ab,kw OR 'e-health':ti,ab,kw OR 'digital health':ti,ab,kw OR 'health apps':ti,ab,kw OR 'remote monitoring':ti,ab,kw OR 'wearable health technology':ti,ab,kw OR 'virtual health':ti,ab,kw OR 'health information technology':ti,ab,kw OR 'digital health

	interventions':ti,ab,kw OR (('health'/exp OR 'health' OR 'health s' OR 'healthful' OR 'healthfulness' OR 'healths') AND 'it solutions':ti,ab,kw)
8	health technology assessment':ti,ab,kw OR 'hta':ti,ab,kw OR 'health technology evaluation':ti,ab,kw OR 'digital health assessment':ti,ab,kw OR 'health interventions':ti,ab,kw OR (('effect' OR 'effective' OR 'effectively' OR 'effectiveness' OR 'effects') AND 'digital tools':ti,ab,kw) OR (('health'/exp OR 'health' OR 'healthful' OR 'healthfulness') AND 'tech' AND 'evaluations':ti,ab,kw) OR 'economic evaluation':ti,ab,kw OR 'impact assessment':ti,ab,kw OR 'medical technology assessment':ti,ab,kw
9	**#5 OR #6 OR #7 OR #8 OR #9**
10	standard care':ti,ab,kw OR 'usual care':ti,ab,kw OR 'conventional care':ti,ab,kw OR 'routine care':ti,ab,kw OR 'current care practices':ti,ab,kw OR 'traditional care':ti,ab,kw
11	traditional care models':ti,ab,kw OR 'non integrated care':ti,ab,kw OR ('non-digital' AND 'care':ti,ab,kw) OR ('usual' AND 'models of care':ti,ab,kw) OR ('non-coordinated' AND 'care':ti,ab,kw) OR ('conventional' OR 'conventionals' AND 'treatment models':ti,ab,kw)
12	**#11 OR #12**
13	caregivers':ti,ab,kw OR 'family caregivers':ti,ab,kw OR 'informal caregivers':ti,ab,kw OR 'primary caregivers':ti,ab,kw OR 'dementia caregivers':ti,ab,kw OR 'unpaid caregivers':ti,ab,kw OR 'caregiving':ti,ab,kw OR 'caregiver burden':ti,ab,kw OR 'caregiving challenges':ti,ab,kw
14	healthcare professionals':ti,ab,kw OR 'nurses':ti,ab,kw OR 'doctors':ti,ab,kw OR 'physicians':ti,ab,kw OR 'social workers':ti,ab,kw OR 'healthcare providers':ti,ab,kw OR 'care managers':ti,ab,kw OR 'geriatric specialists':ti,ab,kw OR 'healthcare staff':ti,ab,kw OR 'dementia specialists':ti,ab,kw OR 'medical professionals':ti,ab,kw
15	**#14 OR #15**
16	quality of care':ti,ab,kw OR 'healthcare quality':ti,ab,kw OR 'patient care quality':ti,ab,kw OR 'healthcare services quality':ti,ab,kw OR 'quality indicators':ti,ab,kw OR 'patient satisfaction':ti,ab,kw OR 'care standards':ti,ab,kw OR 'treatment quality':ti,ab,kw
17	patient outcomes':ti,ab,kw OR 'health outcomes':ti,ab,kw OR 'treatment outcomes':ti,ab,kw OR 'patient health':ti,ab,kw OR 'recovery outcomes':ti,ab,kw OR 'morbidity':ti,ab,kw OR 'mortality':ti,ab,kw OR 'functional outcomes':ti,ab,kw OR 'quality of life':ti,ab,kw
18	healthcare efficiency':ti,ab,kw OR 'care efficiency':ti,ab,kw OR 'healthcare productivity':ti,ab,kw OR 'healthcare service efficiency':ti,ab,kw OR 'cost-effectiveness':ti,ab,kw OR 'service delivery efficiency':ti,ab,kw OR 'health system efficiency':ti,ab,kw OR 'care resource utilization':ti,ab,kw
19	service integration':ti,ab,kw OR 'care coordination':ti,ab,kw OR 'healthcare integration':ti,ab,kw OR 'service delivery':ti,ab,kw OR 'multi level integration':ti,ab,kw OR 'service integration models':ti,ab,kw OR 'healthcare service integration':ti,ab,kw OR 'cross sector integration':ti,ab,kw
20	barriers and facilitators':ti,ab,kw OR 'implementation challenges':ti,ab,kw OR 'barriers to care':ti,ab,kw OR (('obstacle' OR 'obstacles') AND 'care integration':ti,ab,kw) OR 'enablers':ti,ab,kw OR 'facilitators':ti,ab,kw OR 'implementation facilitators':ti,ab,kw OR ('care' AND 'implementation barriers':ti,ab,kw)
21	**#17 OR #18 OR #19 OR #20 OR #21**
22	rct':ti,ab,kw OR 'randomized controlled trial':ti,ab,kw OR 'cohort study':ti,ab,kw OR 'systematic review':ti,ab,kw OR 'meta-analysis':ti,ab,kw OR 'clinical trial':ti,ab,kw OR 'clinical guideline':ti,ab,kw OR 'policy report':ti,ab,kw OR 'qualitative study':ti,ab,kw OR 'mixed methods study':ti,ab,kw
23	**#4 AND #10 AND #13 AND #16 AND #22 AND #23**

**CINAHL Search Strategy**	
Line	Search terms

Search strategy: Care Pathways for Dementia and Neurodegenerative Disorders

1	(MH "Dementia+") OR (MH "Alzheimer's Disease+") OR TI "dementia" OR AB "dementia" OR TI "cognitive decline" OR AB "cognitive decline" OR TI "memory loss" OR AB "memory loss" OR TI "neurocognitive disorders" OR AB "neurocognitive disorders" OR TI "early onset dementia" OR AB "early onset dementia" OR TI "progressive cognitive impairment" OR AB "progressive cognitive impairment"
2	(MH "Neurodegenerative Diseases+") OR TI "Parkinson's disease" OR AB "Parkinson's disease" OR TI "Huntington's disease" OR AB "Huntington's disease" OR TI "Amyotrophic Lateral Sclerosis" OR AB "Amyotrophic Lateral Sclerosis" OR TI "ALS" OR AB "ALS" OR TI "neurodegenerative" OR AB "neurodegenerative" OR TI "motor neuron disease" OR AB "motor neuron disease" OR TI "Lewy body dementia" OR AB "Lewy body dementia" OR TI "frontotemporal dementia" OR AB "frontotemporal dementia"
3	**2 OR 3**
4	TI "evidence-based care pathways" OR AB "evidence-based care pathways" OR TI "clinical pathways" OR AB "clinical pathways" OR TI "care models" OR AB "care models" OR TI "structured care" OR AB "structured care" OR TI "healthcare pathways" OR AB "healthcare pathways" OR TI "integrated care pathways" OR AB "integrated care pathways" OR TI "evidence-based care models" OR AB "evidence-based care models" OR TI "care frameworks" OR AB "care frameworks" OR TI "multidisciplinary care" OR AB "multidisciplinary care"
5	TI "clinical pathways" OR AB "clinical pathways" OR TI "treatment pathways" OR AB "treatment pathways" OR TI "care protocols" OR AB "care protocols" OR TI "management pathways" OR AB "management pathways" OR TI "diagnostic pathways" OR AB "diagnostic pathways" OR TI "standard care plans" OR AB "standard care plans"
6	TI "integrated care" OR AB "integrated care" OR TI "integrated healthcare" OR AB "integrated healthcare" OR TI "coordinated care" OR AB "coordinated care" OR TI "collaborative care" OR AB "collaborative care" OR TI "person centered care" OR AB "person centered care" OR TI "holistic care" OR AB "holistic care" OR TI "multi disciplinary care" OR AB "multi disciplinary care" OR TI "team based care" OR AB "team based care" OR TI "patient centered integrated care" OR AB "patient centered integrated care" OR TI "care integration" OR AB "care integration"
7	TI "digital health technologies" OR AB "digital health technologies" OR TI "telemedicine" OR AB "telemedicine" OR TI "telehealth" OR AB "telehealth" OR TI "mobile health" OR AB "mobile health" OR TI "e-health" OR AB "e-health" OR TI "digital health" OR AB "digital health" OR TI "health apps" OR AB "health apps" OR TI "remote monitoring" OR AB "remote monitoring" OR TI "wearable health technology" OR AB "wearable health technology" OR TI "virtual health" OR AB "virtual health" OR TI "health information technology" OR AB "health information technology" OR TI "digital health interventions" OR AB "digital health interventions"
8	TI "health technology assessment" OR AB "health technology assessment" OR TI "HTA" OR AB "HTA" OR TI "health technology evaluation" OR AB "health technology evaluation" OR TI "digital health assessment" OR AB "digital health assessment" OR TI "health interventions" OR AB "health interventions" OR (TI "effect*" OR AB "effect*") AND (TI "digital tools" OR AB "digital tools") OR TI "economic evaluation" OR AB "economic evaluation" OR TI "impact assessment" OR AB "impact assessment" OR TI "medical technology assessment" OR AB "medical technology assessment"
9	**5 OR 6 OR 7 OR 8 OR 9**
10	TI "standard care" OR AB "standard care" OR TI "usual care" OR AB "usual care" OR TI "conventional care" OR AB "conventional care" OR TI "routine care" OR AB "routine care" OR TI "current care practices" OR AB "current care practices" OR TI "traditional care" OR AB "traditional care"
11	TI "traditional care models" OR AB "traditional care models" OR TI "non integrated care" OR AB "non integrated care" OR (TI "non-digital" AND AB "care") OR (TI "usual" AND AB "models of care") OR (TI "non-coordinated" AND AB "care") OR (TI "conventional" AND AB "treatment models")

12	**11 OR 12**
13	TI "caregivers" OR AB "caregivers" OR TI "family caregivers" OR AB "family caregivers" OR TI "informal caregivers" OR AB "informal caregivers" OR TI "primary caregivers" OR AB "primary caregivers" OR TI "dementia caregivers" OR AB "dementia caregivers" OR TI "unpaid caregivers" OR AB "unpaid caregivers" OR TI "caregiving" OR AB "caregiving" OR TI "caregiver burden" OR AB "caregiver burden" OR TI "caregiving challenges" OR AB "caregiving challenges"
14	TI "healthcare professionals" OR AB "healthcare professionals" OR TI "nurses" OR AB "nurses" OR TI "doctors" OR AB "doctors" OR TI "physicians" OR AB "physicians" OR TI "social workers" OR AB "social workers" OR TI "healthcare providers" OR AB "healthcare providers" OR TI "care managers" OR AB "care managers" OR TI "geriatric specialists" OR AB "geriatric specialists" OR TI "healthcare staff" OR AB "healthcare staff" OR TI "dementia specialists" OR AB "dementia specialists" OR TI "medical professionals" OR AB "medical professionals"
15	**14 OR 15**
16	TI "quality of care" OR AB "quality of care" OR TI "healthcare quality" OR AB "healthcare quality" OR TI "patient care quality" OR AB "patient care quality" OR TI "healthcare services quality" OR AB "healthcare services quality" OR TI "quality indicators" OR AB "quality indicators" OR TI "patient satisfaction" OR AB "patient satisfaction" OR TI "care standards" OR AB "care standards" OR TI "treatment quality" OR AB "treatment quality"
17	TI "patient outcomes" OR AB "patient outcomes" OR TI "health outcomes" OR AB "health outcomes" OR TI "treatment outcomes" OR AB "treatment outcomes" OR TI "patient health" OR AB "patient health" OR TI "recovery outcomes" OR AB "recovery outcomes" OR TI "morbidity" OR AB "morbidity" OR TI "mortality" OR AB "mortality" OR TI "functional outcomes" OR AB "functional outcomes" OR TI "quality of life" OR AB "quality of life"
18	TI "healthcare efficiency" OR AB "healthcare efficiency" OR TI "care efficiency" OR AB "care efficiency" OR TI "healthcare productivity" OR AB "healthcare productivity" OR TI "healthcare service efficiency" OR AB "healthcare service efficiency" OR TI "cost-effectiveness" OR AB "cost-effectiveness" OR TI "service delivery efficiency" OR AB "service delivery efficiency" OR TI "health system efficiency" OR AB "health system efficiency" OR TI "care resource utilization" OR AB "care resource utilization"
19	TI "service integration" OR AB "service integration" OR TI "care coordination" OR AB "care coordination" OR TI "healthcare integration" OR AB "healthcare integration" OR TI "service delivery" OR AB "service delivery" OR TI "multi level integration" OR AB "multi level integration" OR TI "service integration models" OR AB "service integration models" OR TI "healthcare service integration" OR AB "healthcare service integration" OR TI "cross sector integration" OR AB "cross sector integration"
20	TI "barriers and facilitators" OR AB "barriers and facilitators" OR TI "implementation challenges" OR AB "implementation challenges" OR TI "barriers to care" OR AB "barriers to care" OR (TI "obstacle*" OR AB "obstacle*") AND (TI "care integration" OR AB "care integration") OR TI "enablers" OR AB "enablers" OR TI "facilitators" OR AB "facilitators" OR TI "implementation facilitators" OR AB "implementation facilitators" OR (TI "care" AND AB "implementation barriers")
21	**17 OR 18 OR 19 OR 20 OR 21**
22	TI "RCT" OR AB "RCT" OR TI "randomized controlled trial" OR AB "randomized controlled trial" OR TI "cohort study" OR AB "cohort study" OR TI "systematic review" OR AB "systematic review" OR TI "meta-analysis" OR AB "meta-analysis" OR TI "clinical trial" OR AB "clinical trial" OR TI "clinical guideline" OR AB "clinical guideline" OR TI "policy report" OR AB "policy report" OR TI "qualitative study" OR AB "qualitative study" OR TI "mixed methods study" OR AB "mixed methods study"
23	**4 AND 10 AND 13 AND 16 AND 22 AND 23**

Web of Science Search Strategy	
**Line**	**Search Terms**
1	TS=(dementia OR "Alzheimer's disease" OR "cognitive decline" OR "memory loss" OR "neurocognitive disorders" OR "early-onset dementia" OR "progressive cognitive impairment")
2	TS=("neurodegenerative disorder*" OR "Parkinson's disease" OR "Huntington's disease" OR "amyotrophic lateral sclerosis" OR ALS OR neurodegenerative OR "motor neuron disease" OR "Lewy body dementia" OR "frontotemporal dementia")
3	**#2 OR #3**
4	TS=("evidence-based care pathway*" OR "clinical pathway*" OR "care model*" OR "structured care" OR "healthcare pathway*" OR "integrated care pathway*" OR "evidence-based care model*" OR "care framework*" OR "multidisciplinary care")
5	TS=("clinical pathway*" OR "treatment pathway*" OR "care protocol*" OR "management pathway*" OR "diagnostic pathway*" OR "standard care plan*")
6	TS=("integrated care" OR "integrated healthcare" OR "coordinated care" OR "collaborative care" OR "person-centered care" OR "holistic care")
7	TS=("digital health technolog*" OR telemedicine OR telehealth OR "mobile health" OR e-health OR "digital health" OR "health app*" OR "remote monitoring" OR "wearable health technolog*" OR "virtual health" OR "health information technology" OR "digital health intervention*" OR "health IT solution*")
8	TS=("health technology assessment" OR HTA OR "health technology evaluation" OR "digital health assessment" OR health intervention* OR effectiveness of digital tool* OR health tech evaluation* OR "economic evaluation" OR "impact assessment" OR "medical technology assessment")
9	**#5 OR #6 OR #7 OR #8 OR #9**
10	TS=("standard care" OR "usual care" OR "conventional care" OR "routine care" OR current care practice* OR "traditional care")
11	TS=(traditional care model* OR "non-integrated care" OR "non-digital care" OR "usual model* of care" OR "non-coordinated care" OR conventional treatment model*)
12	**#11 OR #12**
13	TS=(caregiver* OR family caregiver* OR informal caregiver* OR primary caregiver* OR dementia caregiver* OR unpaid caregiver* OR caregiving OR "caregiver burden" OR caregiving challenge*)
14	TS=(healthcare professional* OR nurse* OR doctor* OR physician* OR social worker* OR healthcare provider* OR care manager* OR geriatric specialist* OR "healthcare staff" OR dementia specialist* OR medical professional*)
15	**#14 OR #15**
16	TS=("quality of care" OR "healthcare quality" OR "patient care quality" OR "healthcare services quality" OR quality indicator* OR "patient satisfaction" OR care standard* OR "treatment quality")
17	TS=(patient outcome* OR health outcome* OR treatment outcome* OR "patient health" OR recovery outcome* OR morbidity OR mortality OR functional outcome* OR "quality of life")

18	TS=("healthcare efficiency" OR "care efficiency" OR "healthcare productivity" OR "healthcare service efficiency" OR cost-effectiveness OR "service delivery efficiency" OR "health system efficiency" OR "care resource utilization")
19	TS=("service integration" OR "care coordination" OR "healthcare integration" OR "service delivery" OR "multi-level integration" OR service integration model* OR "healthcare service integration" OR "cross-sector integration")
20	TS=("barriers and facilitators" OR "implementation challenges" OR "barriers to care" OR "obstacles to care integration" OR enablers OR facilitators OR "implementation facilitators" OR "care implementation barriers")
21	**#17 OR #18 OR #19 OR #20 OR #21**
22	TS=(RCT OR "randomized controlled trial" OR "cohort study" OR "systematic review" OR "meta-analysis" OR "clinical trial" OR "clinical guideline" OR "policy report" OR "qualitative study" OR "mixed methods study")
23	**#4 AND #10 AND #13 AND #16 AND #22 AND #23**
